# Targeted RNA sequencing with touch imprint cytology samples for non‐small cell lung cancer patients

**DOI:** 10.1111/1759-7714.13460

**Published:** 2020-05-05

**Authors:** Katsutoshi Seto, Katsuhiro Masago, Shiro Fujita, Masataka Haneda, Yoshitsugu Horio, Toyoaki Hida, Hiroaki Kuroda, Waki Hosoda, Ken‐ichi Okubo

**Affiliations:** ^1^ Department of Pathology and Molecular Diagnostics Aichi Cancer Center Nagoya Japan; ^2^ Department of Thoracic Surgery Tokyo Medical and Dental University Tokyo Japan; ^3^ Thoracic Oncology Aichi Cancer Center Nagoya Japan; ^4^ Thoracic Surgery Aichi Cancer Center Nagoya Japan

**Keywords:** Next‐generation sequencing, non‐small cell lung cancer, NGS, RNA

## Abstract

**Background:**

RNA‐based sequencing is considered ideal for detecting pathogenic fusion‐genes compared to DNA‐based assays and provides valuable information about the relative expression of driver genes. However, RNA from formalin‐fixed paraffin‐embedded tissue has issues with both quantity and quality, making RNA‐based sequencing difficult in clinical practice. Analyzing stamp‐derived RNA with next‐generation sequencing (NGS) can address the above‐mentioned obstacles. In this study, we validated the analytical specifications and clinical performance of our custom panel for RNA‐based assays on the Ion Torrent platform.

**Methods:**

To evaluate our custom RNA lung panel, we first examined the gene sequences of RNA derived from 35 NSCLC tissues with diverse backgrounds by conventional methods and NGS. Next, we moved to the clinical phase, where clinical samples (all stamp‐derived RNA) were used to examine variants. In the clinical phase we conducted an NGS analysis while simultaneously applying conventional approaches to assess the feasibility and validity of the panel in clinical practice.

**Results:**

In the prerun phase, all of the variants confirmed with conventional methods were detected by NGS. In the clinical phase, a total of 80 patients were enrolled and 80 tumor specimens were sequenced from February 2018 to December 2018. There were 66 cases in which the RNA concentration was too low to be measured, but sequencing was successful in the vast majority of cases. The concordance between NGS and conventional methods was 95.0%.

**Conclusions:**

RNA extraction using stamp specimens and panel sequencing by NGS were considered applicable in clinical settings.

**Key points:**

**Significant findings of the study**

Next‐generation sequencing using RNA from stamp specimens was able to detect driver gene changes in non‐small cell lung cancer including fusion genes with the same accuracy as conventional methods.

**What this study adds**

Using RNA from stamp specimens obtained from biopsy increases the number of candidate cases for RNA sequencing in clinical settings.

## Introduction

Various targeted agents have been developed for non‐small cell lung cancer (NSCLC), and fusion gene evaluation (eg, *ALK*, *ROS1* and *NTRK*) is now recommended as a standard test for the evaluation of advanced NSCLC.^1^, ^2^ An RNA‐based assay is considered ideal for detecting pathogenic fusion genes compared with a DNA‐based assay, as cancer‐forming driver fusion genes undergo transcription more efficiently than nonfunctioning passenger fusion genes.^3^ Furthermore, for some types of driver mutations (eg, activating *EGFR* mutations), RNA‐based sequencing provides valuable information concerning the relative expression of driver genes.

One serious concern in clinical practice is the need to analyze various driver gene alterations using biopsy specimens, which often contain only a small amount of the malignant component. In addition, the degeneration of RNA inevitably occurs through the process of routine formalin fixation and paraffin embedding. Another concern is that many genetic loci need to be tested simultaneously, and the number of genetic changes to investigate is still increasing.

Stamp cytology specimen can be prepared without much effort during the on‐site microscopic inspection of biopsy specimens to determine whether or not a sufficient amount of tumor cells has been obtained. In our institute, tumor RNA is extracted from the stamp slides for diagnostic purposes. Targeted massively parallel sequencing as a method of examining many gene loci at once provides comprehensive information regarding multiple genetic markers and is currently being implemented in clinical practice. Analyzing stamp‐derived RNA using massive parallel sequencing can address the above‐mentioned obstacles in the management of NSCLC.

In the present study, we validated the analytical specifications and clinical performance of our custom panel for an RNA‐based assay on the Ion Torrent platform. We compared the results with those obtained by the gold‐standard methods for mutational analyses: conventional polymerase chain reaction (PCR) and Sanger sequencing. Furthermore, a highly sensitive PCR method (the cycleave PCR method and the fragment method) was used to verify the results obtained by both conventional PCR and the Ion Torrent platform.

## Methods

### Ethics

This study was approved by the Aichi Cancer Center Hospital's Institutional Review Board. All patients provided their written, informed consent. The study was conducted in accordance with the ethical principles of the Declaration of Helsinki.

### Patient information

Tumor samples were collected from Aichi Cancer Center Hospital, Japan. The current study consisted of two phases; a prerun phase and a clinical phase. The aim of the first stage (prerun phase) was to evaluate the accuracy of the custom panels. For the validation of the custom RNA lung panel, we examined the gene sequences of RNA derived from 35 NSCLC tissues with diverse backgrounds by conventional methods and NGS. Next, we moved to the next clinical phase, where clinical samples (all stamp‐derived RNA) were used to examine variants. In the clinical phase we conducted an NGS analysis while simultaneously applying conventional approaches to assess the feasibility and validity of the panel in clinical practice. A total of 80 patients were enrolled in the clinical phase of the study from February 2018 to December 2018.

### Tissue samples and RNA isolation

RNA used in the prerun phase was derived from various samples, including formalin‐fixed paraffin‐embedded (FFPE) sections and stamp‐slides. Normal tissue was not analyzed. For FFPE sections, tumor sections (surgical, biopsy or cell‐block specimens) were microscopically dissected, deparaffinized with xylene and washed twice in ethanol. After drying at 37°C, the samples were digested with proteinase K at 56°C. A total of three stamp slides were used. RNA extraction was performed applying the RNeasy Mini Kit (Qiagen), as per the manufacturer's instructions. RNA was quantified with the Qubit 2.0 using the RNA HS Assay Kit (Thermo Fisher Scientific). The lowest detectable concentration was 4 ng/μL.

**Figure 1:**
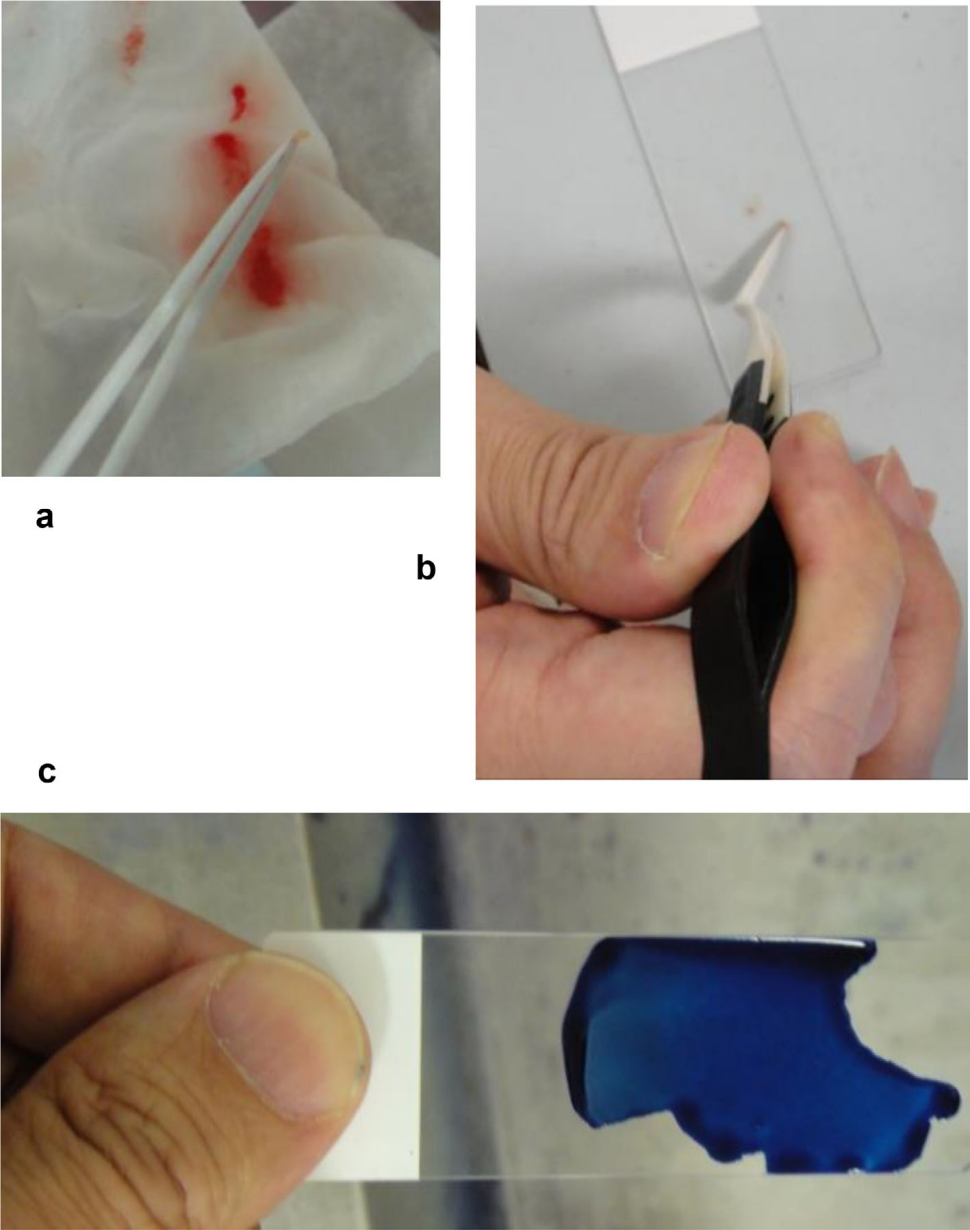
We adopted the direct smear method to obtain stamp slides for cytological examination. (**a**) Once the sample is obtained (**b**) press directly onto slides with forceps to make four stamp slides. (**c**) May‐Giemsa staining should then be performed as quickly as possible. Surgical gloves were not worn in the photographs to facilitate understanding of the procedure,

In the clinical phase, a total of four stamp‐slides were made using the direct smear method, and one slide was subjected to May‐Giemsa staining to confirm the presence of cancer cells (Fig 1). When approximately 100 malignant cells were confirmed by the initial staining, the remaining three slides were used for RNA extraction with the methods described above.

### Conventional PCR and Sanger sequencing

After the synthesis of cDNA, exons 18 to 21 of *EGFR* were amplified by PCR and analyzed. The primers and cycling conditions for PCR amplification were modified from methods published previously.^4^ Sequencing reactions were electrophoresed on an ABI PRISM 3100 (Thermo Fisher Scientific). Direct sequencing of the PCR products was carried out in both sense and antisense directions.

### Cycleave PCR method and the fragment method

We used a chimeric DNA‐RNA‐DNA probe labeled with a fluorescent dye and quencher at each end.^5^ The RNA sequence of the probes corresponds to that of the wild‐type and point mutation (Exon 21 L858R, Exon 21 L861Q) labeled with FAM and ROX, respectively. When mutant molecules are present in the sample and PCR‐amplified DNA generates a complete hybrid with the RNA portion of the mutant probe, RNase‐H digests the probe at the RNA‐DNA heteroduplex into two pieces, leading to a significant increase in fluorescence intensity by the separation of the fluorescent dye from the quencher.

To detect the deletion in exon 19 of the EGFR gene, a common fragment analysis was used. Sample DNA was amplified with a FAM‐labeled primer set, and PCR products were electrophoresed. PCR amplified the shorter segment of DNA, creating a new peak in an electropherogram when a deletion mutation was present.^5^

### Custom panel

A custom panel for lung cancer (Ion Ampliseq 84 328 675–1) was designed by a research team at Thermo Fisher Scientific and manufactured as single primer pools by Thermo Fisher Scientific. This panel covers somatic mutation hot spots in five cancer‐associated genes and is capable of detecting gene translocation events (Table 1), including *MET* exon 14 skipping. To confirm the presence of a sufficient number of reads in the NGS assay, primers for five housekeeping genes (*HMBS*, *ITGB7*, *LMNA*, *MYC*, and *TBP*) were included in the primer mix, and the resultant reads were mapped. Even if no fusion genes were found, the assay itself was considered successful when a sufficient number of reads originating from these housekeeping genes was mapped.

**Table 1:** Assay targets of the custom panel

Hot spot mutation	
*EGFR*, *BRAF*, *KRAS*, *NRAS*, *ERBB2*	
Splicing alteration	
*MET* (exon 14)	
Gene rearrangement (fusion partners)	
*ALK*	: *EML4*, *KIF5B*, *KLC1*, *HIP1*, *TPR*
*ROS1*	: *CD74*, *SDC4*, *SLC34A2*, *EZR*, *TPM3*, *LRIG3*, *GOPC*
*NRG1*	: *CD74*
*RET*	: *KIF5B*, *CUX1*, *CCDC6*

*ALK*, anaplastic lymphoma receptor tyrosine kinase; *BRAF*, V‐raf murine sarcoma viral oncogene homolog b1; *CCDC6*, coiled‐coil domain containing 6; *CUX1*, cut‐like homeobox 1; *EGFR*, epidermal growth factor receptor; *EML4*, echinoderm microtubule associated protein like 4; *ERBB2*, V‐erb‐b2 avian erythroblastic leukemia viral oncogene homolog 2; *EZR*, ezrin; *GOPC*, golgi associated PDZ and coiled‐coil motif containing; *HIP1*, Huntingtin interacting protein 1; *KIF5B*, kinesin family member 5b; *KLC1*, kinesin light chain 1; *KRAS*, V‐Ki‐Ras2 kirsten rat sarcoma 2 viral oncogene homolog; *LRIG3*, leucine rich repeats and immunoglobulin like domains 3; *MET,* MET proto‐oncogene, receptor tyrosine kinase; *NRAS*, neuroblastoma ras viral oncogene homolog; *NRG1*, neuregulin 1; *ROS1*, c‐ros oncogene‐1; *RET*, proto‐oncogene tyrosine‐protein kinase receptor Ret; *SDC4*, syndecan 4; *SLC34A2*, solute carrier family 34 member 2; *TPM3*, tropomyosin 3; *TPR*, translocated promoter region, nuclear basket protein.

In addition, primers designed to amplify the 5′ and 3′ regions of *ALK*, *ROS1*, *RET* and *NRG1* were included in the custom panel. A disproportionately high expression of the 3′ end of the gene compared to the 5′ end implied oncogenic fusion events involving the gene.

### Ion Torrent library preparation and sequencing

In the validation phase, a minimum of 10 ng of total RNA was converted to cDNA using the SuperScript VILO cDNA Synthesis Kit (Thermo Fisher Scientific). In the clinical phase, when the analysis was successfully performed by the conventional method, we conducted an NGS assay. An Ion Torrent adapter‐ligated library was generated using the custom panel described above. The concentration and size of the library were determined using the Applied Biosystems StepOne Real‐Time PCR system and Ion Library TaqMan Quantitation kit (both from Thermo Fisher Scientific). Sample emulsion PCR, emulsion breaking and enrichment were performed using the Ion Chef system (Thermo Fisher Scientific), according to the manufacturer's instructions. An input concentration of one DNA template copy per ion sphere particle (ISP) was added to the emulsion PCR master mix, and the emulsion was generated using the Ion Chef System (Thermo Fisher Scientific). Template‐positive ISPs were enriched, and sequencing was performed using semiconductor chips on the Ion Torrent S5 (Thermo Fisher Scientific). In this study, we used semiconductor chips of Ion 530 Chip Kit, which was later we changed to the Ion 540 Chip Kit, in order to collect an adequate number of sequenced reads.

### Variant calling

Data from the S5 runs were initially processed using the Ion Torrent platform‐specific pipeline software program Torrent Suite v4.0 (Thermo Fisher Scientific) to generate sequence reads, trim adapter sequences and filter and remove poor signal‐profile reads. We prepared a reference sequence to match the custom primer and mapped reads to the reference using Torrent Suite. The total number of reads mapped is an indicator of whether or not the NGS run was successful. The total mapped panel reads were counted, and 20 000 reads was considered sufficient for a further analysis; however, with a small sample size, clinical samples sometimes fail to achieve that level. In such cases, we proceeded with the analysis and searched for variants.

Initial variant calling from the Ion AmpliSeq sequencing data was generated with a plug‐in “variant caller” program. To eliminate erroneous base calling, three filtering steps were used to generate final variant calling. The first filter was set at an average depth of total coverage of >50, each variant coverage of >15 and *P* < 0.01. The second filter was employed by visually examining mutations using the CLC Genomics Workbench software program version 10.0.0 (Qiagen), as well as by filtering out possible strand‐specific errors.

### DNA‐based NGS

In the detection of driver gene alteration other than gene rearrangement, DNA‐based NGS was performed only when discrepancies occurred in the results with conventional methods and RNA‐based sequencing. We used AmpliSeq Cancer Hotspot panel to validate the genetic alterations, described previously.^6^ Briefly, FFPE samples were deparaffinized and DNA isolated from the sections using the QIAamp DNA Mini Kit (Qiagen). We created a library with Ion AmpliSeq Library Kit 2.0 and AmpliSeq Cancer Hotspot panel, as per the manufacturer's instructions. Subsequent sequencing and variant calling were the same as RNA‐based sequencing.

## Results

### Prerun phase

We examined 35 NSCLC cases, of which 23 had known rearrangements confirmed by fluorescence in situ hybridization (FISH) and immunohistochemistry; 11 cases were *ROS1* rearranged, eight *ALK* rearranged, and four were *RET* rearranged NSCLC (Table 2). All cases were previously confirmed by FISH and immunohistochemistry. Five cases were revealed to have *MET*‐skipping variants using conventional methods, and another five cases had tumors containing either *EGFR*, *KRAS* or *BRAF* gene mutations. The remaining two cases were judged to be driver‐mutation negative.

**Table 2:** Clinical characteristics of the patients enrolled in the validation phase

Gender	
Male	15
Female	20
Age	
Mean age	59.1
Age range	29–84
Smoking status	
Never	20
Ever	15
Histology	
Adenocarcinoma	29
Squamous cell carcinoma	3
NOS	2
Pleomorphic carcinoma	1
Specimen obtained	
Operation	18
Stamp cytology	12
Cell block	5
Primary or metastatic	
Primary	25
Metastatic	10

NOS, not otherwise specified.

The clinical characteristics of the evaluated cases are shown in Table 3. Because the rearrangement patterns of some cases were rare, we validated the rearrangement not only with touch imprint stamp samples; 18 cases were analyzed with surgically resected samples and 12 cases of touch imprint stamp samples were tested, and the rest of five cases we used RNA derived from cell‐block.

**Table 3:** Overview of the variants analyzed in the validation phase

ALK rearrangement	
*EML4*‐*ALK*	8
**ROS1** **rearrangement**	
*CD74*‐*ROS1*	6
*SDC4*‐*ROS1*	4
*EZR*‐*ROS1*	1
**RET rearrangement**	
*KIF5B*‐*RET*	3
*CCDC6*‐*RET*	1
**MET exon skipping**	
Exon14 skipping	5
***EGFR*** ** mutation**	
Exon 19 deletion and exon 20 Thr790Met	1
Exon 21 Leu858Arg and exon 20 Thr790Met	1
Exon 20 Ser768Ile	1
***KRAS*** ** mutation**	
Exon 2 Gly13Cys	1
***BRAF*** ** mutation**	
Exon 15 Val600Glu	1
**No major driver alterations**	2

*ALK*, anaplastic lymphoma receptor tyrosine kinase; *BRAF*, V‐raf murine sarcoma viral oncogene homolog b1; *CCDC6*, coiled‐coil domain containing 6; *EGFR*, epidermal growth factor receptor; *EML4*, echinoderm microtubule associated protein like 4; *EZR*, ezrin; *KIF5B*, kinesin family member 5b; *KRAS*, V‐Ki‐Ras2 kirsten rat sarcoma 2 viral oncogene homolog; *MET*, MET proto‐oncogene, receptor tyrosine kinase; *RET*, proto‐oncogene tyrosine‐protein kinase receptor Ret; *SDC4*, syndecan 4.

In the prerun phase, the median number of leads was 595 910, ranging from 3183 to 1 710 173. All of the variants confirmed with conventional methods were detected by NGS.

### Clinical phase

Next, we started the clinical phase. From February 2018 to December 2018, a total of 80 patients were enrolled, and 80 tumor specimens were sequenced. The clinical characteristics are shown in Table 4. All RNA samples were derived from stamp‐cytology. There were 66 cases where the RNA concentration was too low to be measured (less than 4 ng/μL) and 14 cases were the value was measurable (mean value 10.78, range 4.0–23.0 ng/μL). Because of the rarity of ROS1 or RET‐rearranged NSCLC, there were no patients who developed tumors with such rearrangement during the period (Table 5). The median number of NGS reads was 1 432 555, ranging from 3034 to 5 371 225 and 76 of the 80 samples achieved a total read count of 20 000.

**Table 4:** Clinical characteristics of the patients enrolled in the clinical phase

Gender	
Male	45
Female	35
Age	
Mean age	64.9
Age range	38–90
Smoking status	
Never	28
Ever	51
Unknown	1
Histology	
Adenocarcinoma	60
Squamous cell carcinoma	6
NOS	11
Large	1
Pleomorphic carcinoma	1
Sarcomatoid carcinoma	1
Primary or metastatic	
Primary	51
Metastatic	29

NOS, not otherwise specified.

**Table 5:** Overview of the variants analyzed in the clinical phase

ALK rearrangement	
*EML4*‐*ALK*	2
*KIF5B*‐*ALK*	1
*KLC1*‐*ALK*	1
**NRG1 rearrangement**	
*CD74*‐*NRG* *1*	1
**MET exon skipping**	
Exon14 skipping	3
***EGFR*** ** mutation**	
Exon 19 deletion	15
Exon 21 Leu858Arg	4
Exon 19 deletion and exon 20 Thr790Met	3
Exon 21 Leu858Arg and exon 20 Thr790Met	2
Exon 18 Gly719Ala	2
Exon 18 Gly719Ser	1
Exon 20 Ser768Ile	1
***KRAS*** ** mutation**	
Exon 2 Gly12Asp	4
Exon 2 Gly12Cys	2
Exon 2 Gly12Ala	1
Exon 2 Gly13Glu	1
***BRAF*** ** mutation**	
Exon 15 Val600Glu	1
***ERRB2*** ** mutation**	
Exon 20 insertion	1
**No major driver alterations**	34

*ALK*, anaplastic lymphoma receptor tyrosine kinase; *BRAF*, V‐raf murine sarcoma viral oncogene homolog b1; *EGFR*, epidermal growth factor receptor; *EML4*, echinoderm microtubule associated protein like 4; *ERBB2*, V‐erb‐b2 avian erythroblastic leukemia viral oncogene homolog 2; *KIF5B*, kinesin family member 5b; *KLC1*, kinesin light chain 1; *KRAS*, V‐Ki‐Ras2 kirsten rat sarcoma 2 viral oncogene homolog; *MET*, MET proto‐oncogene, receptor tyrosine kinase; *NRG1*, neuregulin 1.

The concordance between NGS and conventional methods was 95.0%. Of the four inconsistencies (Table 6), a reanalysis with DNA‐based NGS supported the results of conventional methods in the first case (exon 19 deletion in *EGFR*). The deletion was not detectable by RNA‐based NGS and was only detected by DNA‐based NGS. Although this case had the *EGFR*‐activating mutation, the maximum effect of afatinib was stable disease, lasting as short as three months, so some biological mechanisms other than the EGFR pathway may have contributed to the cancer progression. This failure of RNA‐based NGS may be related to the paucity of EGFR mRNA with the activating mutation, considering that the EGFR‐tyrosine kinase inhibitor afatinib showed a minor effect on tumors in the patient. In the second case, although a very weak signal was detected by the cycleave method, the *KRAS* status was determined to be wild‐type. On NGS (both RNA‐based and DNA‐based), *KRAS* Gly13Glu was detected. In the third case, *KRAS* Gly12Cys was also negative by the conventional cycleave method but was detected by NGS (both RNA‐based and DNA‐based). From the stamp‐derived RNA in the fourth case, the *CD74*‐*NRG1* fusion gene was detected only by NGS (RNA‐based) and later was confirmed using cDNA‐based Sanger sequencing.

**Table 6:** Summary of discordances between NGS and conventional methods for variant detection

	Number of cases
NGS (+) / conventional (+)	76
NGS (+) / conventional (−)	3
NGS (−) / conventional (+)	1

NGS, next‐generation sequencing.

## Discussion

Fusion gene detection is mandatory for the initial evaluation of advanced NSCLC, as various agents that target fusion gene kinase (eg, *ALK* or *ROS1*) have proven effective against fusion gene‐derived tumors and been approved. Given their ability to detect structural rearrangements and provide information about the expression, most groups have favored the use of RNA‐based approaches for fusion gene detection. However, one major concern associated with the efficient examination of gene rearrangement is the technical difficulty of obtaining high‐quality RNA.

RNA extraction from FFPE samples, especially small biopsy specimens, is often difficult. Even after extraction, formalin‐induced RNA alteration and fragmentation is inevitable. In order to obtain high‐quality RNA while minimizing sample loss, it is desirable to obtain RNA from fresh tissue, and using stamp cytology specimens is an ideal option for such an approach.

NGS from cytologic specimens has been frequently reported in thyroid cancer. Fine‐needle aspiration cytology is the most common approach to evaluate thyroid nodules pathologically; however, 20% to 30% of the results are indeterminable. Most of the patients with indeterminable results undergo diagnostic surgery, which is unnecessary if the thyroid nodule proves to be benign. Due to the need to complement cytologic examination, NGS has been used to comprehensively search for genetic abnormalities associated with thyroid cancer. Because thyroid cancer sometimes shows gene rearrangement, RNA‐based NGS has been performed. Several groups have reported the result of the cytologic specimen‐derived RNA‐based NGS assay, which provided both high sensitivity and specificity for cancer detection in clinical settings.^7^, ^8^, ^9^ Guseva *et al*. reported that the NGS assay using RNA from cytologic specimens was extended to thyroid and lung cancers, and that the results were completely consistent with the results using RNA from FFPE specimens.^9^ However, there were only 17 lung cancer cases, including small cell lung cancer. Our study had more participants than the studies described above, and is the largest panel sequence data from stamp cytology specimens of NSCLC patients to our knowledge.

In the present study, even if the concentration of RNA was low (below the measurement sensitivity), RNA could be reverse‐transcribed into cDNA, and the resultant cDNA was successfully tested with NGS. Previously, Miki *et al*. reported the utility of serosal stamp cytology‐derived DNA samples for molecular analyses.^10^ They extracted RNA for serosal stamp cytology specimens obtained from patients with gastric cancer and subsequently analyzed the samples by reverse transcription‐PCR to determine the levels of carcinoembryonic antigen (CEA) and cytokeratin 20 (CK20). They successfully identified patients at a high risk for peritoneal recurrence after curative surgery for gastric cancer. With stamp‐derived RNA, a genetic analysis can be carried out if the samples are handled appropriately.

In some cases, DNA‐based sequencing is still required to assess the tumor mutational burden (TMB). The TMB was recently shown to contribute to the immune recognition of cancer and was suggested to be a predictor of the response to cancer immunotherapy.^11^, ^12^, ^13^ Several reports have indicated that the TMB is generally low in driver mutation‐positive cases, which is considered to be one of the reasons why the response rate of immune‐checkpoint inhibitors is inferior to that in driver‐mutation negative cases.^13^, ^14^ Information on driver gene mutations should be obtained first in RNA‐based sequence analyses. Then, only in cases where the driver gene mutation is negative, comprehensive large‐panel DNA‐based NGS testing should be performed to determine the profile of genetic mutations related to carcinogenesis, which is important for deciding on entry into clinical trials, as well as TMB information.

When applying the method described above, it is necessary to carefully examine the handling flow of biomaterial at the facility. For example, a rapid on‐site cytologic examination is a critical step that needs to be performed before NGS. At the NGS stage, library preparation and chip loading are both conducted by the Ion Chef semi‐automatically, which helps smooth the transition to the resultant bioinformatic tasks.

One major limitation associated with our system is that previous knowledge of all possible fusion patterns is necessary in order to prepare an ideal panel for examination. To overcome this limitation, this assay includes a method for detecting *ALK*, *ROS1*, *RET* and *NRG1* fusion genes based on the ratio of the 3′ end and 5′ end expression. A disproportionally high expression of the 3′ end of the gene compared to the 5′ end implies an oncogenic fusion event involving the gene.^15^ If an abnormally high expression of 3′ ends is detected in the expression pattern, the application of alternative methods should be considered (eg, FISH or rapid amplification of cDNA ends method).

Another limitation associated with our study is that we were unable to evaluate all rearrangements using NGS with RNA from stamp cytology because of the rarity of some gene rearrangements (*ROS1* and *RET*). *NTRK* oncogenic fusion is an emerging target for the subset of solid malignancies including NSCLC. Several clinical trials have shown the safety and efficacy of TRK fusion kinase inhibitors, and highly potent pan‐TRK inhibitors have already been approved by regulatory agencies.^16^, ^17^, ^18^, ^19^ We are currently designing a panel that includes new detection primers for the *NTRK* fusion gene.

Lastly, the best biomaterial for RNA‐based clinical sequencing was not determined in our study. We chose RNA extraction from stamp cytology to preserve as much tissue as possible, in addition, to avoid highly‐degenerated specimens. However, many facilities use FFPE tissue after confirming that tumor cells are abundant. Whether the imprint sample is more suitable for genetic testing than conventional samples (tissue block) is unknown from the study. Moreover, assuming that a touch imprint sample is used, it is unknown how many slides are best used for RNA extraction. The larger the number, the more RNA can be extracted, but the handling on site becomes complicated.

The vast majority of current diagnostic genomic practices are being challenged by massive parallel sequencing technology. Panel‐based RNA assays as a tool for the initial genomic evaluation of known driver gene alteration can deliver useful results that compete with existing traditional methods in both feasibility and accuracy.

## Disclosure

The authors report no conflict of interest.
